# Co-Administration of Gagam-Sipjeondaebo-Tang and Ibuprofen Alleviates the Inflammatory Response in MPTP-Induced Parkinson’s Disease Mouse Model and RAW264.7 Macrophages

**DOI:** 10.3390/pathogens10030268

**Published:** 2021-02-26

**Authors:** Sodam Won, Jade Heejae Ko, Hayoung Jeon, Seong-Sik Park, Seung-Nam Kim

**Affiliations:** 1 College of Korean Medicine, Dongguk University, Goyang 10326, Korea; sodam.won@dongguk.ac.kr (S.W.); heejae.ko@dongguk.ac.kr (J.H.K.); hayoung.jeon@dongguk.ac.kr (H.J.); 2 Graduate School, Dongguk University, Seoul 04620, Korea; 3 Department of Sasang Constitutional Medicine, Dongguk University, Goyang 10326, Korea; seongsik.park@dongguk.edu

**Keywords:** Parkinson’s disease, neuroinflammation, herb, Gagam-Sipjeondaebo-Tang, ibuprofen

## Abstract

Parkinson’s disease (PD), a common neurodegenerative disease, is characterized by degeneration of dopaminergic neurons with neuroinflammation. Gagam-Sipjeondaebo-Tang (GST), a traditional herbal formula made of twelve medicinal herbs, is known to be effective in PD, and the use of ibuprofen has been associated with a lower risk of PD. The aim of this study was to evaluate whether the combined administration of GST and ibuprofen affects the inflammatory response of Parkinson’s disease. MPTP-induced parkinsonian mouse models were treated with GST or ibuprofen using oral gavage once a day for 5 days. The effects of GST were examined by measuring the TH level and expression of CD68 in the mice brain in addition to behavioral tests. The anti-inflammatory effect of GST on the LPS-treated RAW264.7 murine macrophages was examined using the NO assay. Inflammatory cytokines were analyzed using quantitative-PCR and flow cytometry. In the results, GST significantly improved the loss of dopaminergic neurons and alleviated PD-induced behavioral deficits. GST also decreased macrophage activation in the MPTP-induced PD mouse model. Interestingly, co-administration of GST and ibuprofen showed a synergistic effect in improving the loss of dopaminergic neurons and decreasing the activation of macrophages. Moreover, the NO level decreased in LPS-stimulated macrophages with this combined treatment. GST reduced iNOS, COX-2, IL-1β, and IL-6 levels, and co-administration with ibuprofen showed a synergistic effect. Furthermore, pretreatment of GST reduced the expression levels of MCP-1 and IL-12 p70 in LPS-stimulated RAW264.7 cells. These results can possibly suggest a future therapeutic approach for PD patients.

## 1. Introduction

Parkinson’s disease (PD) is a common neurodegenerative disease characterized by a substantial loss of dopaminergic neurons in the substantia nigra (SN) pars compacta. Multiple cellular and molecular mechanisms, including mitochondrial dysfunction and oxidative stress, might contribute to neuronal death [1,2]. Many mechanisms have been suggested to explain the etiology of PD, and it has been postulated that neuroinflammation is involved in the progressive loss of dopaminergic neurons and development of PD [3,4]. As inflammatory changes are a cause of PD, the effect of anti-inflammatory drugs on PD has been widely studied regarding the alleviation of neuroinflammation [5,6]. The use of nonsteroidal anti-inflammatory drugs (NSAIDs), especially ibuprofen, has been shown to be associated with a reduced risk of PD in previous studies [7,8,9]. However, long-term use of NSAIDs has side effects such as peptic ulcer, stroke, and acute hemodynamic kidney damage [10]. Yet, there is insufficient evidence to suggest ibuprofen as a reliable pharmacological treatment option for PD patients.

Traditional Chinese medicines have positive effects on PD via neuroprotection and neuroinflammation [11,12]. Gagam-Sipjeondaebo-Tang (GST) is a novel herbal formula composed of twelve medicinal herbs derived from Sipjeon-daebo-tang (SDT). GST was created by adding two herbs to SDT: Uncaria rhynchophylla and Gastrodia elata Blume. Both of these herbs have been reported to have a positive effect on PD [13,14] and have been used in East Asian countries for patients with movement disorders. Furthermore, Uncaria rynchophyella extract exerts anti-PD effects through neuroprotective effects on induced toxicity in PC12 cells and 6-hydroxydopamine-induced rats [15], and by inhibition of heat shock protein 90, which is implicated in PD pathology induced by α-synuclein [14]. SDT, a traditional herbal medicine, is effective in activating the immune response and is widely used to treat nervous system diseases [16]. SDT was also found to reduce inflammation in a mouse model of muscle atrophy by reducing inflammatory cytokines [17]. In previous studies, GST inhibited neuronal death activation in a PD mouse model [18], but the effect of GST on inflammation has not been well studied. Moreover, co-administration of GST with conventional drugs has not been well studied.

The aim of this study was to investigate the effect of co-administration of GST with ibuprofen on neuroinflammation in a PD mouse model and lipopolysaccharide (LPS)-stimulated RAW264.7 cells.

## 2. Results

### 2.1. Effects of Co-Administration of GST and Ibuprofen on the TH Level in SN and ST of a PD Mouse Model

**Figure 1:**
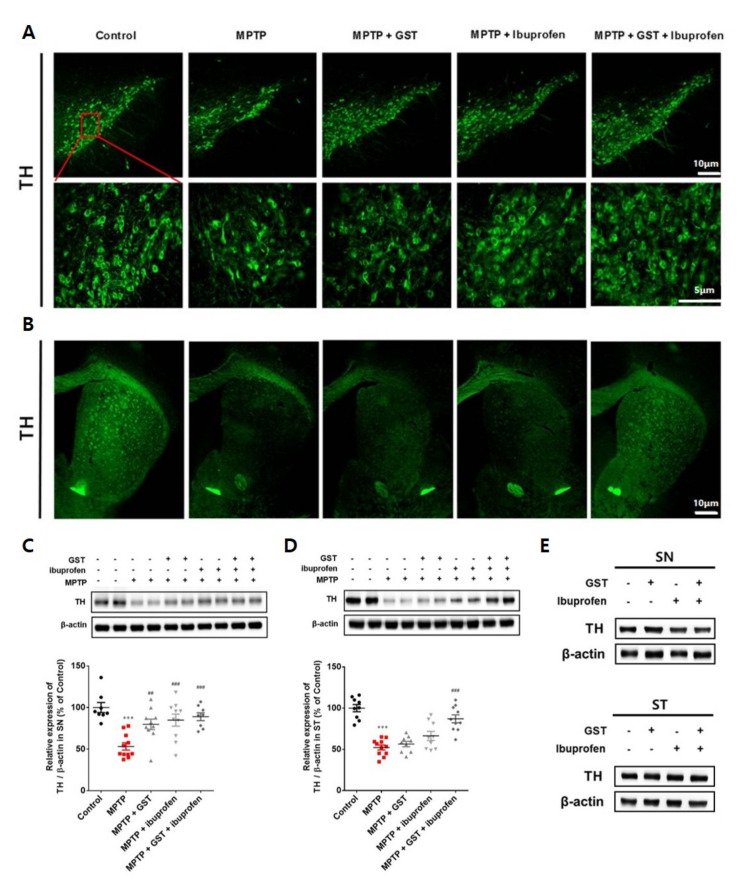
Effects of ibuprofen and Gagam-Sipjeondaebo-Tang (GST) on tyrosine hydroxylase (TH) level in substantia nigra (SN)/ striatum (ST) of 1-methyl-4-phenyl-1,2,3,6-tetrahydropyridine (MPTP)-induced Parkinson’s disease (PD) Mice. Mice were administered with MPTP and ibuprofen, GST, or both GST + ibuprofen (n = 9–10 each group). Immunohistochemistry of TH in the (**A**) SN (scale bars indicate 5 and 10 μm) and (**B**) ST (scale bars indicate 10 μm). Western blot for TH expression of (**C**) SN and (**D**) ST of MPTP-induced PD mice. Control mice were treated with ibuprofen, GST, or GST + ibuprofen. Western blot for TH expression of (**E**) SN and ST of control mice. *** *p* < 0.001, significant difference as compared to the control; ## *p* < 0.01, ### *p* < 0.001, significance difference as compared to MPTP.

To demonstrate that the GST extract and ibuprofen in the present experiments were effective in improving the tyrosine hydroxylase (TH) level in the striatum (ST) and SN, an immunohistochemistry and a western blot were performed. As a result of the analysis, the number of TH-positive neurons in the SN and ST of the 1-methyl-4-phenyl-1,2,3,6-tetrahydropyridine (MPTP) injection mice was significantly reduced compared to the control group. Compared with the MPTP group, the TH level in the SN of the drug-administered group was increased, and in the ST of the GST + ibuprofen group, the TH level was significantly increased than that of the GST group and the ibuprofen group (Figure 1A,B). Western blotting Analyses showed that there were significant decreases in TH-positive neurons in the TH in the SN (53.0 ± 4.4 vs. control, *p* < 0.01) and ST (52.0 ± 3.0 vs. control, *p* < 0.01) of MPTP-administered mice. The TH level increased in the SN of GST, ibuprofen, and GST + ibuprofen groups (80.0 ± 6.2, 85.0 ± 7.3, and 89.0 ± 4.7, respectively vs. MPTP, *p* < 0.01). Changes in the TH level in ST of GST and ibuprofen groups were not statistically significant; however, there was a significant increase in the TH level in the ST of GST + ibuprofen group (87.0 ± 4.8 vs. MPTP, *p* < 0.001). Importantly, we found evidence of the potential synergic effect of GST and ibuprofen on both ST and SN of MPTP-administered mice (Figure 1C,D). There was no significant difference on TH level when GST, ibuprofen, or GST+ibuprofen was treated on control mice (Figure 1E).

### 2.2. Effects of Co-Administration of GST and Ibuprofen on the Motor Function Impairment in PD Mouse Model

**Figure 2:**
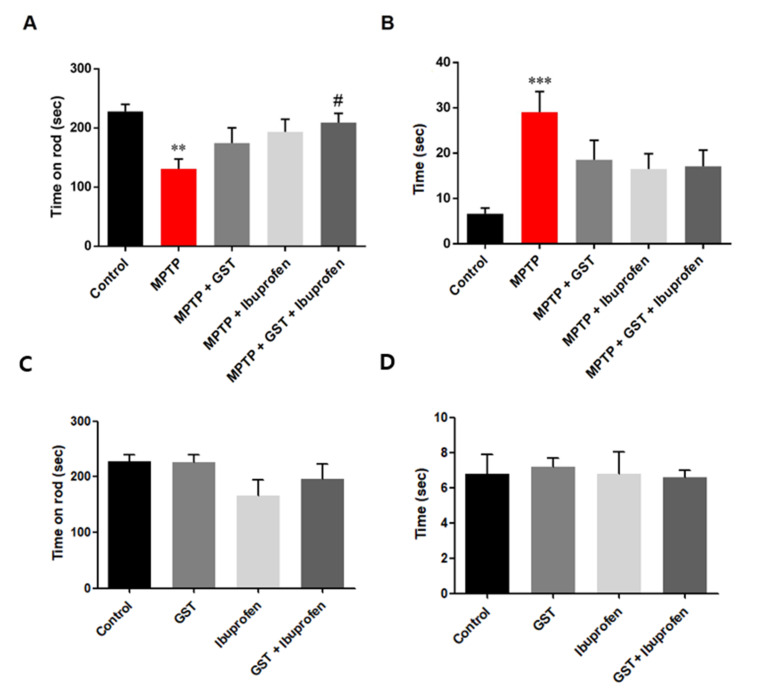
Behavior assessment of MPTP-induced PD mice. Mice were administered with MPTP and ibuprofen, GST, or both GST + ibuprofen were treated (n = 9–10 each group). (**A**) Motor performance of MPTP-induced PD mice was measured in the rotarod-test. (**B**) Motor performance of MPTP-induced PD mice was assessed in the pole test. Control mice were treated with ibuprofen, GST, or both GST+ibuprofen. (**C**) Motor performance of control mice was measured in the rotarod-test. (**D**) Motor performance of control mice was assessed in the pole test. Measured the time before the mouse reached the ground on the pole. ** *p* < 0.01, *** *p* < 0.001, significant difference as compared to the control, # *p <* 0.05, significant difference as compared to MPTP.

Rotarod test is often used to evaluate the motor function in MPTP-induced PD mouse model. Compared to the control group, mice with MPTP injections significantly reduced latency. This shows that MPTP resulted in motor impairment in mice. However, the positive effect of GST was also seen in behavior assessments. Compared to the MPTP group, mice in the GST and GST + ibuprofen groups showed increase in their locomotor activity (*p* < 0.01 control versus MPTP and *p* < 0.05 MPTP versus MPTP + GST + ibuprofen, Figure 2A). On the last day of drug administration (day 5), pole test was performed to examine the effect on the improvement of dysfunction in MPTP-induced PD mice. As a result of the pole test, the total time to the floor was significantly increased compared to the control group after MPTP injection (*p* < 0.001). This means that the PD mouse, induced by MPTP, has a motor dysfunction (Figure 2B). There was no significant difference on motor function when GST, ibuprofen, or GST+ibuprofen was treated on control mice (Figure 2C,D).

### 2.3. Effects of Co-Administration of GST on Expression of Macrophage in a PD Mice Brain

**Figure 3:**
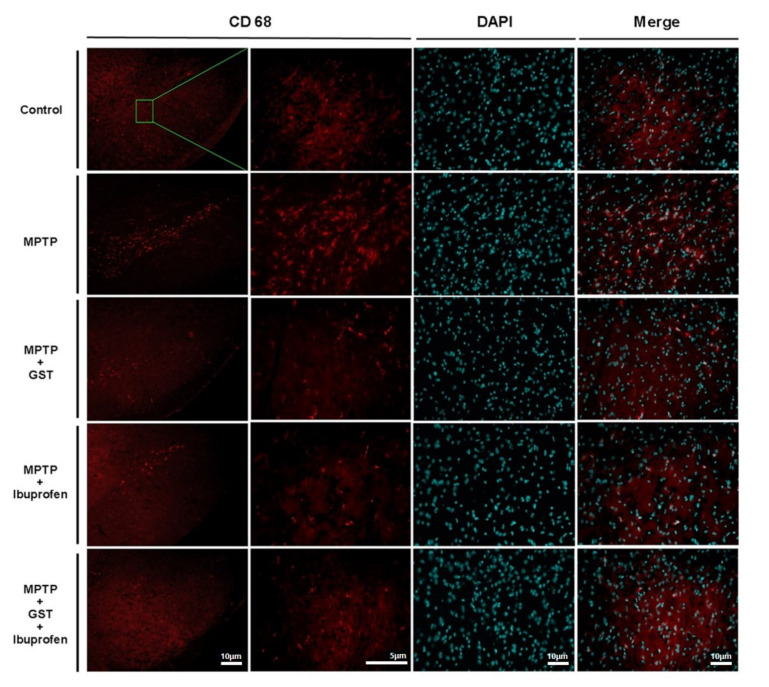
Effects of GST on MPTP-induced neuronal inflammation. Immunohistochemistry staining for CD68 in SN of MPTP-induced PD mouse model. Magnified images demonstrate the localization of CD68-positive macrophages in SN. The CD68 expressions are significantly higher in MPTP mice compared with controls. The CD68 expressions are decreased in MPTP + GST, MPTP + ibuprofen, and MPTP + GST + ibuprofen group (scale bars indicate 5 and 10 μm).

To examine changes of macrophage-mediated inflammation in PD mice brain, we used CD68 which is a type 1 transmembrane glycoprotein expressed by macrophages. Immunofluorescence results showed that CD68 in SN was highly expressed in the MPTP group but significantly decreased in expression in the GST, ibuprofen, and GST + ibuprofen groups (Figure 3).

### 2.4. Effects of Co-Administration of GST and Ibuprofen on LPS-Stimulated Nitric Oxide Secretion

**Figure 4:**
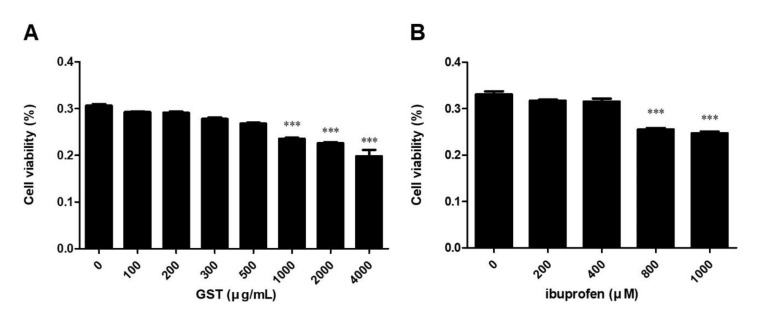
Measurement of cell viability in RAW264.7 cells. Cell viability was measured using MTT assay. Cells were treated with different concentrations of (**A**) GST (from 100 to 4000 µg/mL) and (**B**) ibuprofen (from 200 to 1000 µM). Results are mean ± SEM of three independent experiments: *** *p* < 0.05, in comparison to the control.

After detecting an increase in CD68 in SN, we used RAW264.7 cells, a macrophage cell line, for further investigation of the inflammatory response at the cellular level. RAW264.7 cells were treated with different concentrations of GST (from 100 to 4000 µg/mL) and/or ibuprofen (from 200 to 1000 µM) for 24 h. The cell viability level significantly reduced in concentrations of 1000, 2000, and 4000 µg/mL of GST (Figure 4A) and in 800 and 1000 µM of ibuprofen (Figure 4B). Therefore, we used doses of 200 and 400 µM of ibuprofen and 250 and 500 µg/mL of GST for further experiments.

**Figure 5:**
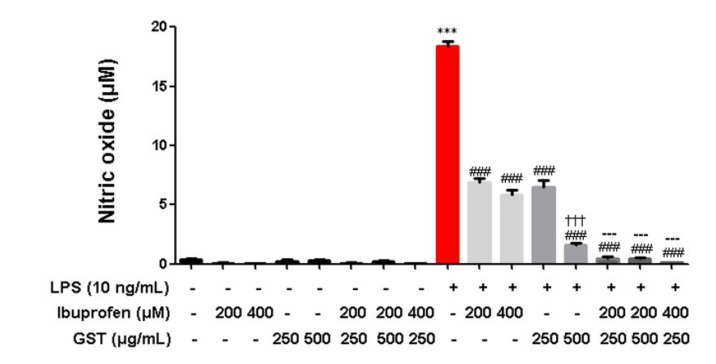
Effects of GST on LPS-stimulated nitric oxide secretion. RAW264.7 cells were treated with various concentrations of GST. The cells were treated with 200 and 400 µM of ibuprofen or 250 and 500 µg/mL of GST. Cells were stimulated with 10 ng/mL of LPS for 24 h. The collected supernatants were reacted with Griess reagent, and the absorbance level was measured at 570 nm. Results are mean ± SEM of three independent experiments; *** *p* < 0.05 in comparison to the control; ### *p* < 0.001. in comparison to the LPS; ††† *p* < 0.05, in comparison to the LPS + GST 250 ug/mL; --- *p* < 0.05, in comparison to the LPS + GST 500 µg/mL.

Subsequently, we compared the ibuprofen and GST groups. The nitric oxide (NO) level increased after LPS stimulation (18.0 ± 0.4 compared to control, *p* < 0.05). Administering 200 and 400 µM of ibuprofen to the LPS-treated RAW 264.7 cells decreased the NO level (6.9 ± 0.4 and 5.8 ± 0.4, respectively compared to the LPS-treated group, *p* < 0.05), and 400 µM of ibuprofen resulted in a further reduction of the NO secretion level. In addition, 250 and 500 µg/mL of GST also reduced the NO secretion levels in a dose-dependent manner (6.5 ± 0.5 and 1.6 ± 0.1, respectively compared to the LPS-treated group, *p* < 0.05). Significantly, administration of 250 µg/mL of GST plus 200 and 400 µM of ibuprofen reduced the NO secretion to the control level (0.5 ± 0.1 and 0.1 ± 0.0 compared to the ibuprofen and GST-treated groups, respectively, *p* < 0.05) (Figure 5).

### 2.5. Effects of Co-Administration of GST and Ibuprofen on Inflammatory Cytokines in LPS-Stimulated RAW 264.7 Cells

**Figure 6:**
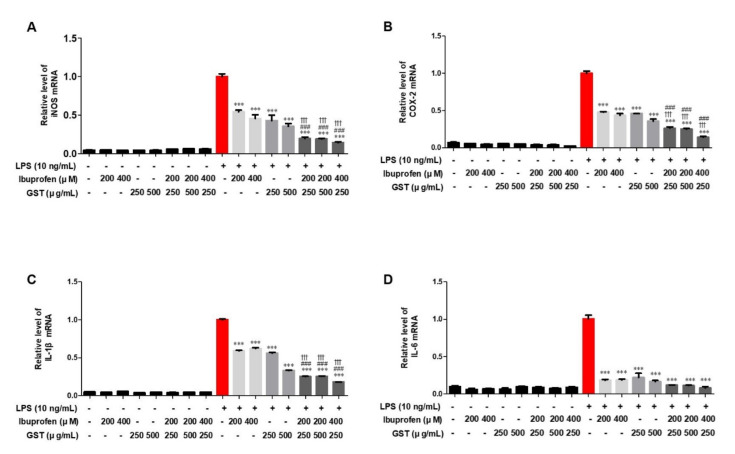
Effects of GST and ibuprofen on iNOS, COX-2, IL-1β, and IL-6 in RAW264.7. Measurement of pro-inflammatory cytokines. Effect of GST or ibuprofen on LPS-stimulated (**A**) iNOS, (**B**) COX-2, (**C**) IL-1β, and (**D**) IL-6 RNA expression in RAW264.7 cells by Q-PCR. Cells were pre-treated with various concentrations of GST or ibuprofen for 30 min and then stimulated with LPS (10 ng/mL) for 24 h. The cells with or without LPS group were pretreated with GST, ibuprofen and ibuprofen + GST. GAPDH was used as the housekeeping gene, and the indicated values represent three independent experiments. Results are mean ± SEM of triplicate experiments: ††† *p* < 0.05, in comparison with the 200 µM ibuprofen + LPS group; ### *p* < 0.001, in comparison with the 400 µM ibuprofen + LPS group.

As a result of quantitative polymerase chain reaction, the expression level of inflammatory cytokines was increased in LPS-stimulated RAW264.7 cells compared to the control group. After each inflammatory cytokine level reached the maximum value with LPS treatment to the cells, each inflammatory cytokine, iNOS (Figure 6A), COX-2 (Figure 6B), IL-1β (Figure 6C), and IL-6 (Figure 6D), significantly decreased when different doses of ibuprofen and GST were administered. It was evident that treatment with both ibuprofen and GST resulted in a synergic effect in reducing the number of inflammatory cytokines.

**Figure 7:**
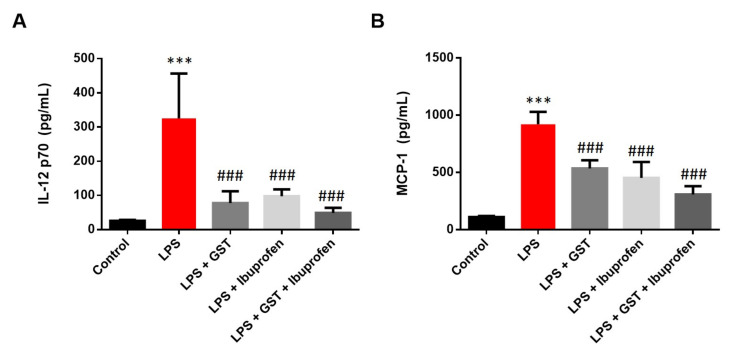
Measurement of representative cytometric bead array on IL-12p70 and MCP-1 in RAW264.7 cells. The graph shows cytokine levels in (**A**) IL-12p70 and (**B**) MCP-1. RAW 264.7 cells were pretreated with 500 µg/mL GST, 200 µM ibuprofen, or combined treatment for 30 min, and then stimulated with LPS (10 ng/mL). Culture protein extracts of each group were analyzed by CBA by flow cytometry. Results are mean ± SEM (n = 3); *** *p* < 0.001, in comparison with the Control group; ### *p* < 0.001, in comparison with the LPS group.

To determine the effect of GST and combined treatment in eliciting an immune response, a cytometric bead array test was performed to evaluate the cytokine IL-12p70 and monocyte chemoattractant protein-1 (MCP-1) release of RAW264.7 cells stimulated with LPS. Upon LPS activation in RAW264.7 cells, IL-12p70 and MCP-1(Figure 7A,B) showed a maximum increase in cytokine production (compared to the control group, *p* < 0.001). However, GST, ibuprofen, or combined pre-treatment significantly inhibited this LPS-induced cytokine production (compared to the LPS group, *p* < 0.001).

## 3. Discussion

Neuroinflammation is a major cause of PD. To explore the therapeutic effects of GST on PD, we used MPTP-induced PD animal model and LPS-stimulated macrophage cells which are most widely used models as much as 6-OHDA model and human induced pluripotent stem cells-based model [19,20].

In the PD mouse model, GST and ibuprofen administration alleviated MPTP-induced motor impairment. Loss of TH activity (due to a decrease in TH protein level) may be the cause of dopamine deficiency [21]. Consistent with these data, the MPTP-induced PD mouse model also exhibited dopaminergic neuronal degeneration with decreased TH-positive cells in the SN. However, GST and ibuprofen administration inhibited the decrease in TH-positive cells in the PD mouse model. These results indicate that GST and ibuprofen have therapeutic effects on motor deficits and dopaminergic neuronal degeneration.

In addition, GST alleviated MPTP-induced neuroinflammation by inhibiting activated macrophage. An in vitro study further revealed that GST inhibited macrophage and inflammatory cytokines were significantly reduced in LPS-stimulated RAW264.7 macrophages.

Many reports have provided evidence for pathophysiology of PD, and most recent studies have suggested that neuroinflammation might have a role in the development of PD [22,23]. Activated microglia and macrophages in the SN of a PD mouse model is reliable evidence explaining neuroinflammation in PD. Microglia-derived production of cytotoxic mediators is likely to be involved in mechanisms contributing to neuronal death, which leads to further PD progression [24,25]. In this study, MPTP-induced PD model mice in the SN region showed high expression of macrophage-mediated inflammation. However, treatment with GST and ibuprofen significantly decreased expression of macrophage.

Inflammation sequentially causes an increase in NO, which is a pro-inflammatory mediator [26]. In this study, we found that GST and ibuprofen significantly downregulated the NO level in LPS-stimulated cell lines. Interestingly, the degree of decrease in the GST-treated cell line differed from that in ibuprofen-treated cell lines. More importantly, treatment with GST and ibuprofen significantly reduced NO levels compared to treatment with GST or ibuprofen alone.

Activated microglia and macrophage release large amounts of inflammatory cytokines such as IL-1β, IL-6, which are detrimental to the survival of dopaminergic neurons [27,28,29,30]. IL-1β is produced by activated macrophages, and the induction of COX-2 by IL-1β in the central nervous system (CNS) is known to cause inflammatory pain [31]. IL-6 is secreted by macrophages in response to specific microbial molecules called pathogen-associated molecular pattern (PAMP), and increased expression of IL-6 is observed in neurodegenerative disorders such as PD and Alzheimer’s disease [32]. As cytokines such as IL-1β, IL-6 are involved in the pathogenesis of these diseases, their treatment should involve inhibition of these inflammatory molecules [33,34]. Inflammation plays a central role in the pathogenesis of PD and the resulting immune reactions from surrounding microglia and macrophages [35,36]. Macrophages involved in the inflammatory response are activated by LPS and Toll-like receptor ligands. These are outer membrane components of gram-negative bacteria, which promote the secretion of inflammatory cytokines such as IL-1β, IL-12, and IL-6, thereby, causing a wide range of anti-inflammatory reactions [37,38]. The formation of these mediators causes inflammation by generating excess NO through the expression of iNOS, and COX-2 [39]. MCP-1 is a CC chemokine that stimulates leukocytes and is mostly expressed by macrophages. Its level is associated with pathogenesis of inflammatory diseases and activation of macrophages [40,41]. Therefore, inhibition of aberrant macrophage activation may play an important role in treating inflammatory diseases. In this study, macrophage activation was observed as the levels of cytokines (IL-6, IL-1β, IL-12), inflammatory mediators (COX-2, iNOS), and inflammation-related chemokines (MCP-1) increased in RAW264.7 cells stimulated by LPS. GST effectively reduced the mRNA and protein levels of inflammatory factors. These results confirm that GST is effective in suppressing the inflammatory responses induced by LPS.

Many treatments and medications for PD have been studied in the past decades. Many studies have implicated the inflammatory processes in PD progression in animal models. There is not enough data to draw a final conclusion in explaining synergistic activities between ibuprofen and GST. There are different opinions on using herbal ingredients as a therapeutic method since compounds of the herbal ingredients were not fully understood. For instance, curcumin has been reported as pan-assay-interference compounds and invalid metabolic panacea, thus raising concerns about its medicinal properties [42,43,44]. Several compounds of GST have been found, yet, the effect of each chemical compound still needs further investigation [13,45,46,47,48]. It is necessary to continue further studies in order to provide reliable quality standards of medicinal herbs. Furthermore, it is hoped that based on what this study suggests, it can be a comprehensive evidence to develop neuroinflammation target therapies that can prevent and delay the progression of not only PD but also other neurodegenerative diseases.

In this study, we found protective effects of co-administration of GST and ibuprofen on PD mouse model by down-regulating macrophage expression and elevated inflammatory cytokine level. These results suggest that co-administration of GST and ibuprofen may be an effective alternative treatment for neuroinflammatory diseases such as PD.

## 4. Materials and Methods

### 4.1. Animals and Experiment Design

**Figure 8:**
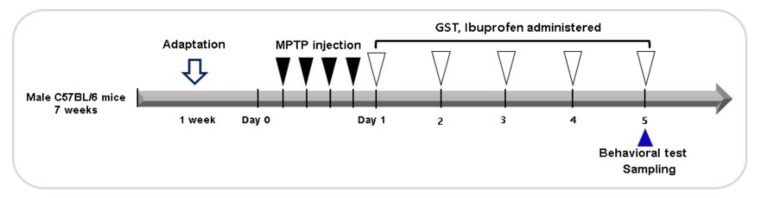
Timeline of the animal experiment design. The filled black arrowheads indicate the MPTP intraperitoneal injection to induce Parkinson’s disease mice. The PD mouse model was induced by intraperitoneal injection of MPTP 20 mg/kg 4 times at 2 h intervals. Empty white arrowheads indicate oral administration of GST (200 mg/kg) and ibuprofen (80 mg/kg). The blue arrow represents the day of the behavior test.

Male C57BL/6 mice (Orient-Bio Co., Seongnam, Korea), 7 weeks of age and weighing 20–25 g each were used in this study. All the animals were kept on a 12/12 h-light cycle in a room with access to food and water ad libitum. All experiments were approved by the Dongguk University Animal Care Committee for animal welfare and maintained in strict accordance with Guidelines (DGU-IACUC-2018-022-2). A total of 50 mice were randomly divided into five groups. All the mice except the control group were intraperitoneally injected with MPTP (20 mg/kg of body weight, Sigma-Aldrich, St. Louis, MO, USA) every 2 h, four times a day in total and control mice were injected with the same volume of saline on the same schedule. Diluted ibuprofen at final concentration of 80 mg/kg was orally administered to mice in the ibuprofen group once a day for five consecutive days and 200 mg/kg of GST was also orally administered to the GST group once a day for five consecutive days. The same volume of saline was given to the control group by oral administration on the same schedule (Figure 8). Experiment groups are as following; control group (administration of saline for 5 day), MPTP group (administration of saline for 5 day, MPTP 20 mg/kg intraperitoneal injections 4 times at 2 h interval), MPTP + GST group (administration of GST 200 mg/kg for 5 day), MPTP + ibuprofen group (administration of ibuprofen 80 mg/kg for 5 day) and MPTP + GST + ibuprofen group (administration of GST 200 mg/kg and ibuprofen 80 mg/kg for 5 day). The dose of all treatments was followed by our previous study [18].

### 4.2. Preparation of GST Extract

GST extracts were composed of twelve herbs (Table 1). The twelve herb components were manufactured by Omniherb (Seoul, Korea) and extracted in 800 mL of 30% ethanol for 4 h. The extracts were filtered through a 40 µm filter paper (Whatman, Kent, UK) and filtrates were concentrated by using a rotary vacuum evaporator (EYELA, Tokyo, Japan) at 80 °C. The concentrates were lyophilized by a freeze dryer (EYELA, Tokyo, Japan) and the yield of lyophilized GST extract was 22.7%.

### 4.3. Motor Function Tests

Mice were tested on the rotarod and pole test a day before and 5 days after MPTP injection. For the pole test, each mouse was positioned head upward near the top of the pole. The time taken to reach the floor was measured by subtracting the time mice reached the floor (P1) by the time when mice completely rotated head downward (P2). The motor coordination of mice was evaluated on a rotarod treadmill. The accelerating speed of the rotarod was set to increase from 5 rpm/min to 30 rpm/min within 4 min. The mice were placed on the treadmill, and the time that each mouse spent on the treadmill was scored as seconds with 240 s as maximum score.

### 4.4. Cell Culture

RAW264.7, a murine macrophage cell line was purchased from the American Type Culture Collection (Rockville, MD, USA). The RAW264.7 cell line was cultured in DMEM (HyClone, Logan, UT, USA) supplemented with 10% Fetal Bovine Serum (FBS) and 1% penicillin/streptomycin (HyClone, Logan, UT, USA) and the cells were cultured at 37 °C in a 5% CO_2_ humidified atmosphere. The cells were subcultured once a day for 2 days.

### 4.5. Cell Viability Assay

3-(4, 5-dimethyl thiazol-2-yl)-2, 5-diphenyltetrazolium bromide (MTT) assay was used to evaluate the cell viability in different concentrations of ibuprofen and GST. The RAW264.7 cells were seeded at 2.5 × 10^5^ cells/well in 24-well plates and incubated for 24 h. After incubation, cells were treated with different concentrations of ibuprofen (from 0 to 1000 µM) and GST (from 0 to 4000 µg/mL) for 24 h. After the pretreatment with ibuprofen and GST, 100 µL of cell viability reagent (Thermo Fisher Scientific, Waltham, MA, USA) was added in each well and incubated for 90 min. Absorbance was measured at a wavelength of 570 nm by using a multimode microplate reader (VICTOR Nivo, Atlanta, GA, USA).

### 4.6. Nitric Oxide Assay

RAW264.7 cells in 24-well, which were seeded for 24 h at a concentration of 2.5 × 10^5^ cell/well were pretreated with various concentrations of ibuprofen (200 µM and 400 µM) and GST (250 µg/mL and 500 µg/mL) for 24 h. The cells were then stimulated with 10 ng/mL LPS (*Escherichia coli* O111:B4; Sigma-Aldrich, St. Louis, MO, USA) for further 24 h. After obtaining the culture supernatant, the NO Plus Detection Kit (Intron Biotech, Daejeon, Korea) in the same amount as the supernatant was added. The mixture of supernatant and NO detection agent was incubated for 20 min at room temperature and its absorbance was measured at a wavelength of 570 nm by using a microplate reader (VICTOR Nivo, Atlanta, GA, USA).

### 4.7. Quantitative Real-Time PCR

RAW264.7 cells were plated at 1 × 10^6^ cells/well in 6-well plates for 24 h. Different concentrations of ibuprofen (200 µM and 400 µM) and GST (250 µg/mL and 500 µg/mL) were pretreated for 30 min. The cells were then stimulated with 10 ng/mL LPS (E.coli O111:B4; Sigma-Aldrich, St. Louis, MO, USA) for further 24 h. RNA was extracted using ReliaPrep™ RNA Miniprep Systems (Promega, Madison, WI, USA). Quantitative real-time PCR (Q-PCR) was performed using FastStart Essential DNA Green Master (Roche Life Science, Germany) with various primers. Real-time reactions were detected by the LightCycler^®^ 96 Instrument. GAPDH was used as a housekeeping gene, and the data were cycle threshold (Ct) value was normalized by relative quantification using the 2 -ΔΔCT method. Sequences used in Q-PCR are as following: IL-6-F; 5’-CTTCTTGGGACTGATG-3’, IL-6-R; 5’-CTGGCTTTGTCTTTCT-3’, IL-1β-F; 5’-GATCCACACTCTCCAGCTGCA-3’, IL-1β-R; 5’-CAACCAACAAGTGATATTCTCCATG-3’, iNOS-F; 5’-GACAAGCTGCATGTGACATC-3’, iNOS-R; 5’-GCTGGTAGGTTCCTGTTGTT-3’, COX-2-F; 5’-TCCAGATCACATTTGATTGA-3’, COX-2-R; 5’-TCTTTGACTGTGGGAGGATA-3’, GAPDH-F; 5’-AACTTTGGCATTGTGGAAGG-3’, GAPDH-R; 5’-ACACATTGGGGGTAGGAACA-3’.

### 4.8. Western Blot

Brain samples were homogenized within 400 µL of T-PER and Halt™ Protease and Phosphatase Inhibitor Cocktail mixture (Thermo Fisher Scientific, Waltham, MA, USA). The homogenized samples were centrifuged at 21,205× *g* for 20 min and supernatants were collected in new tubes. Western blotting was performed to examine expression levels of TH in SN and ST. Thirty micrograms of extracted proteins were separated by 10% SDS-PAGE gel and transferred onto PVDF membrane (Millipore, Burlington, MA, USA). The membranes were blocked with 5% skim milk in TBS-T for 60 min. After blocking, membranes were incubated with primary TH antibody (1:2000; Novus, St. Charles, MO, USA), and β-Actin (1:5000; Novus, St. Charles, MO, USA) overnight and washed 3 times with TBS-T, and incubated with HRP-conjugated goat anti-mouse IgG (H+L) antibody (Novus, St. Charles, MO, USA) for 1.5 h at room temperature. Membranes were then washed 3 times with TBS-T and the proteins were detected with Fusion Solo with Evo6 camera (Vilber Lourmat, Marne-la-Vallée, France).

### 4.9. Cytometric Bead Array

RAW264.7 cells were plated at 1 × 10^6^ cells/well in 6-well plates for 24 h and 30 min pretreated with GST (500 µg/mL). The cells were then stimulated with 10 ng/mL LPS (E.coli O111:B4; Sigma-Aldrich, St. Louis, MO, USA) for further 24 h. The cells were resuspended with RIPA buffer (Thermo Scientific, Waltham, MA, USA) containing protease and phosphatase inhibitor cocktail (Thermo Scientific, Waltham, MA, USA) on ice for 20 min and cells were discarded by centrifugation. Cytometric bead array (CBA) was performed to examine cytokine expression at the protein level in RAW264.7 cells stimulated with LPS. The antibody detected by extracted protein (300 µg) and detection reagent was incubated with Mouse cytometric bead array kit (CBA Mouse Inflammation Kit; BD Bioscience, San Jose, CA, USA) for 2 h in the dark at room temperature. All unbound antibodies were washed (1.0 mL buffer) and then 300 µL retested and measured with a CytoFLEX flow cytometer (Beckman Coulter Inc., Brea, CA, USA).

### 4.10. Immunofluorescence

In an in vivo experiment, after the last behavior tests were completed, mice were sacrificed, and brains were quickly harvested, fixed in 4% paraformaldehyde for 24 h, and kept in 30% sucrose solution at 4 °C until they are sectioned. The brain samples were embedded in an optimal cutting temperature compound (Sakura Finetek, Torrance, CA, USA) for 3 h and frozen sections were cut to a thickness of 20 µm using a cryostat (Thermo Fisher Scientific, Waltham, MA, USA). Frozen sections were blocked with 1% bovine serum albumin (BSA; VWR Life Science, Radnor, PA, USA) in 0.01 M PBS for 1 h at room temperature. After blocking, samples were incubated with rabbit anti-TH primary antibody (1:1000; Novus, St. Charles, MO, USA), and CD-68 (1:50; Thermo Fisher Scientific, Waltham, MA, USA) overnight at room temperature. The samples were washed with PBS (Biosesang, Seoul, Korea) and incubated with goat anti-mouse and goat anti-rabbit IgG (H+L) antibodies, Alexa Fluor 568 (1:2000; Invitrogen, Waltham, MA, USA) for 1.5 h at room temperature. After washing with 0.01M PBS, samples were mounted with Fluoroshield with DAPI (Sigma-Aldrich, St. Louis, MO, USA). All the stained samples were detected by a fluorescence microscope (Olympus, Tokyo, Japan).

### 4.11. Statistics

Experimental results are presented as means ± standard error and data were analyzed by One-way ANOVA followed by Bonferroni’s post hoc multiple comparison test by using GraphPad Prism software (GraphPad Software Inc, San Diego, CA, USA). The differences with *p* < 0.05 was considered statistically significant.

## Figures and Tables

**Table 1:** The compositions of GST.

Latin Name	Scientific Name (Family Name)	Ratio
Ginseng Radix Alba	*Panax ginseng C. A.* Meyer (Araliaceae)	3
Atractylodis Rhizoma Alba	*Atractylodes macrocephala* Koidzumi (Compositae)	2
Hoelen	*Poria cocos* Wolf (Polyporaceae)	2
Glycyrrhizae Radix et Rhizoma	*Glycyrrhiza uralensis* Fischer (Leguminosae)	2
Rehmanniae Radix Preparata	*Rehmannia glutinosa Liboschitz ex* Steudel (Scrophulariaceae)	2
Angelicae Gigantis Radix	*Angelica gigas* Nakai (Umbelliferae)	2
Cnidii Rhizoma	*Cnidium officinale* Makino (Umbelliferae)	2
Paeoniae Radix	*Paeonia lactifora* Pallas (Paeoniaceae)	2
Astragali Radix	*Astragalus membranaceus* Bunge (Leguminosae)	3
Bupleuri Radix	*Bupleurum falcatum Linne* (Umbelliferae)	2
Uncariae Ramulus et Uncus	*Uncaria rhynchophylla* (Rubiaceae)	2
Gastrodiae Rhizoma	*Gastrodia elata* Blume (Orchidaceae)	2

## Data Availability

Not applicable.

## References

[1] Sanchez-Padilla J., Guzman J.N., Ilijic E., Kondapalli J., Galtieri D.J., Yang B., Schieber S., Oertel W., Wokosin D., Schumacker P.T. (2014). Mitochondrial oxidant stress in locus coeruleus is regulated by activity and nitric oxide synthase. Nat. Neurosci..

[2] Ganjam G.K., Bolte K., Matschke L.A., Neitemeier S., Dolga A.M., Höllerhage M., Höglinger G.U., Adamczyk A., Decher N., Oertel W.H. (2019). Mitochondrial damage by α-synuclein causes cell death in human dopaminergic neurons. Cell Death Dis..

[3] Wang Q., Liu Y., Zhou J. (2015). Neuroinflammation in Parkinson’s disease and its potential as therapeutic target. Transl. Neurodegener..

[4] Kicińska H., Wróblewska M. (1966). Preliminary sero-epidemiologic studies on arboviruses in selected occupational groups of the healthy population of the country. Przegl. Epidemiol..

[5] Pisanu A., Lecca D., Mulas G., Wardas J., Simbula G., Spiga S., Carta A.R. (2014). Dynamic changes in pro- and anti-inflammatory cytokines in microglia after PPAR-γ agonist neuroprotective treatment in the MPTPp mouse model of progressive Parkinson’s disease. Neurobiol. Dis..

[6] Rangasamy S.B., Dasarathi S., Pahan P., Jana M., Pahan K. (2019). Low-Dose Aspirin Upregulates Tyrosine Hydroxylase and Increases Dopamine Production in Dopaminergic Neurons: Implications for Parkinson’s Disease. J. Neuroimmune Pharmacol..

[7] Asanuma M., Miyazaki I. (2007). Common anti-inflammatory drugs are potentially therapeutic for Parkinson’s disease?. Exp. Neurol..

[8] Asanuma M., Miyazaki I. (2006). Nonsteroidal anti-inflammatory drugs in Parkinson’s disease: Possible involvement of quinone formation. Expert Rev. Neurother..

[9] Asanuma M., Nishibayashi-Asanuma S., Miyazaki I., Kohno M., Ogawa N. (2001). Neuroprotective effects of non-steroidal anti-inflammatory drugs by direct scavenging of nitric oxide radicals. J. Neurochem..

[10] Li Z., Choi D.-Y., Shin E.-J., Hunter R.L., Jin C.H., Wie M.-B., Kim M.S., Park S.J., Bing G., Kim H.-C. (2008). Phenidone protects the nigral dopaminergic neurons from LPS-induced neurotoxicity. Neurosci. Lett..

[11] Wei R., OuYang J., Lin W., Lin T. (2018). Curative Anti-Inflammatory Properties of Chinese Optimized Yinxieling Formula in Models of Parkinson’s Disease. Evid. Based Complement. Altern. Med..

[12] Wang T., Li C., Han B., Wang Z., Meng X., Zhang L., He J., Fu F. (2020). Neuroprotective effects of Danshensu on rotenone-induced Parkinson’s disease models in vitro and in vivo. BMC Complement. Med. Ther..

[13] Doo A.-R., Kim S.-N., Hahm D.-H., Yoo H.H., Park J.-Y., Lee H., Jeon S., Kim J., Park S.-U., Park H.-J. (2014). Gastrodia elata Blume alleviates L-DOPA-induced dyskinesia by normalizing FosB and ERK activation in a 6-OHDA-lesioned Parkinson’s disease mouse model. BMC Complement. Altern. Med..

[14] Lan Y.-L., Zhou J.-J., Liu J., Huo X.-K., Wang Y.-L., Liang J.-H., Zhao J.-C., Sun C.-P., Yu Z.-L., Fang L.-L. (2018). Uncaria rhynchophylla Ameliorates Parkinson’s Disease by Inhibiting HSP90 Expression: Insights from Quantitative Proteomics. Cell. Physiol. Biochem..

[15] Shim J.S., Kim H.G., Ju M.S., Choi J.G., Jeong S.Y., Oh M.S. (2009). Effects of the hook of Uncaria rhynchophylla on neurotoxicity in the 6-hydroxydopamine model of Parkinson’s disease. J. Ethnopharmacol..

[16] Liu H., Wang J., Sekiyama A., Tabira T. (2008). Juzen-taiho-to, an Herbal Medicine, Activates and Enhances Phagocytosis in Microglia/Macrophages. Tohoku J. Exp. Med..

[17] Ishida T., Iizuka M., Ou Y., Morisawa S., Hirata A., Yagi Y., Jobu K., Morita Y., Miyamura M. (2019). Juzentaihoto Suppresses Muscle Atrophy in Streptozotocin-Induced Diabetic Mice. Biol. Pharm. Bull..

[18] Ko J.H., Lee J.-H., Choi B., Park J.-Y., Kwon Y.-W., Jeon S., Park S.-D., Kim S.-N. (2018). Neuroprotective Effects of Gagam-Sipjeondaebo-Tang, a Novel Herbal Formula, against MPTP-Induced Parkinsonian Mice and MPP+-Induced Cell Death in SH-SY5Y Cells. Evid. Based Complement. Altern. Med..

[19] Jagmag S.A., Tripathi N., Shukla S.D., Maiti S., Khurana S. (2015). Evaluation of Models of Parkinson’s Disease. Front. Neurosci..

[20] Buhidma Y., Rukavina K., Chaudhuri K.R., Duty S. (2020). Potential of animal models for advancing the understanding and treatment of pain in Parkinson’s disease. NPJ Park Dis..

[21] Bae N., Ahn T., Chung S., Oh M.S., Ko H., Oh H., Park G., Yang H.O. (2011). The neuroprotective effect of modified Yeoldahanso-tang via autophagy enhancement in models of Parkinson’s disease. J. Ethnopharmacol..

[22] Lee Y., Lee S., Chang S.-C., Lee J. (2019). Significant roles of neuroinflammation in Parkinson’s disease: Therapeutic targets for PD prevention. Arch. Pharmacal. Res..

[23] Ramkumar M., Rajasankar S., Gobi V.V., Janakiraman U., Manivasagam T., Thenmozhi A.J., Essa M.M., Chidambaram R., Chidambaram S.B., Guillemin G.J. (2018). Demethoxycurcumin, a Natural Derivative of Curcumin Abrogates Rotenone-induced Dopamine Depletion and Motor Deficits by Its Antioxidative and Anti-inflammatory Properties in Parkinsonian Rats. Pharm. Mag..

[24] Blaylock R.L. (2017). Parkinson’s disease: Microglial/macrophage-induced immunoexcitotoxicity as a central mechanism of neurodegeneration. Surg. Neurol. Int..

[25] Marinova-Mutafchieva L., Sadeghian M., Broom L., Davis J.B., Medhurst A.D., Dexter D.T. (2009). Relationship between microglial activation and dopaminergic neuronal loss in the substantia nigra: A time course study in a 6-hydroxydopamine model of Parkinson’s disease. J. Neurochem..

[26] Sharma J.N., Al-Omran A., Parvathy S.S. (2007). Role of nitric oxide in inflammatory diseases. Inflammopharmacology.

[27] Koprich J.B., Reske-Nielsen C., Mithal P., Isacson O. (2008). Neuroinflammation mediated by IL-1beta increases susceptibility of dopamine neurons to degeneration in an animal model of Parkinson’s disease. J. Neuroinflammation.

[28] Qin L., Liu Y., Hong J.-S., Crews F.T. (2013). NADPH oxidase and aging drive microglial activation, oxidative stress, and dopaminergic neurodegeneration following systemic LPS administration. Glia.

[29] Chang R.C., Hudson P., Wilson B., Haddon L., Hong J.-S. (2000). Influence of neurons on lipopolysaccharide-stimulated production of nitric oxide and tumor necrosis factor-α by cultured glia. Brain Res..

[30] Wu D.-C., Teismann P., Tieu K., Vila M., Jackson-Lewis V., Ischiropoulos H., Przedborski S. (2003). NADPH oxidase mediates oxidative stress in the 1-methyl-4-phenyl-1,2,3,6-tetrahydropyridine model of Parkinson’s disease. Proc. Natl. Acad. Sci. USA.

[31] Dinarello C.A., Simon A., Van Der Meer J.W.M. (2012). Treating inflammation by blocking interleukin-1 in a broad spectrum of diseases. Nat. Rev. Drug Discov..

[32] Rothaug M., Becker-Pauly C., Rose-John S. (2016). The role of interleukin-6 signaling in nervous tissue. Biochim. Biophys. Acta (BBA) Bioenerg..

[33] Dinarello C.A. (2011). Interleukin-1 in the pathogenesis and treatment of inflammatory diseases. Blood.

[34] Unver N., McAllister F. (2018). IL-6 family cytokines: Key inflammatory mediators as biomarkers and potential therapeutic targets. Cytokine Growth Factor Rev..

[35] Kim B.-W., Koppula S., Kumar H., Park J.Y., Kim I.W., More S.V., Kim I.-S., Han S.-D., Kim S., Yoon S.-H. (2015). α-Asarone attenuates microglia-mediated neuroinflammation by inhibiting NF kappa B activation and mitigates MPTP-induced behavioral deficits in a mouse model of Parkinson’s disease. Neuropharmacology.

[36] Stephenson J., Nutma E., Van Der Valk P., Amor S. (2018). Inflammation in CNS neurodegenerative diseases. Immunology.

[37] Mosser D.M., Edwards J.P. (2008). Exploring the full spectrum of macrophage activation. Nat. Rev. Immunol..

[38] Liao X., Sharma N., Kapadia F., Zhou G., Lu Y., Hong H., Paruchuri K., Mahabeleshwar G.H., Dalmas E., Venteclef N. (2011). Krüppel-like factor 4 regulates macrophage polarization. J. Clin. Investig..

[39] Xie C., Li X., Zhu J., Wu J., Geng S., Zhong C. (2019). Magnesium isoglycyrrhizinate suppresses LPS-induced inflammation and oxidative stress through inhibiting NF-κB and MAPK pathways in RAW264.7 cells. Bioorg. Med. Chem..

[40] Zhu T., Zhang W., Feng S.-J., Yu H.-P. (2016). Emodin suppresses LPS-induced inflammation in RAW264.7 cells through a PPARγ-dependent pathway. Int. Immunopharmacol..

[41] Zhong Y., Liu T., Lai W., Tan Y., Tian D., Guo Z. (2012). Heme oxygenase-1-mediated reactive oxygen species reduction is involved in the inhibitory effect of curcumin on lipopolysaccharide-induced monocyte chemoattractant protein-1 production in RAW264.7 macrophages. Mol. Med. Rep..

[42] Nelson K.M., Dahlin J.L., Bisson J., Graham J., Pauli G.F., Walters M.A. (2017). The Essential Medicinal Chemistry of Curcumin: Miniperspective. J. Med. Chem..

[43] Baell J., Walters M.A. (2014). Chemistry: Chemical con artists foil drug discovery. Nat. News.

[44] Baker M. (2017). Deceptive curcumin offers cautionary tale for chemists. Nat. Cell Biol..

[45] Yang H.J., Ma J.Y., Weon J.B., Ma C.J. (2011). Simultaneous determination of eight marker compounds in the traditional herbal medicine, Sipjundaebo-tang by HPLC-DAD. Arch. Pharmacal Res..

[46] Oh Y.-C., Cho W.-K., Jeong Y.H., Im G.Y., Yang M.C., Ma J.Y. (2012). Fermentation Improves Anti-Inflammatory Effect of Sipjeondaebotang on LPS-Stimulated RAW 264.7 Cells. Am. J. Chin. Med..

[47] Mita Y., Dobashi K., Shimizu Y., Nakazawa T., Mori M. (2002). Surface expression of toll-like receptor 4 on THP-1 cells is modulated by Bu-Zhong-Yi-Qi-Tang and Shi-Quan-Da-Bu-Tang. Methods Find. Exp. Clin. Pharmacol..

[48] Fujiwara H., Iwasaki K., Furukawa K., Seki T., He M., Maruyama M., Tomita N., Kudo Y., Higuchi M., Saido T.C. (2006). Uncaria rhynchophylla, a Chinese medicinal herb, has potent antiaggregation effects on Alzheimer’s β-amyloid proteins. J. Neurosci. Res..

